# A Curriculum for Lumbar Puncture Training in Internal Medicine Residency

**DOI:** 10.15694/mep.2019.000033.1

**Published:** 2019-02-26

**Authors:** Benjamin Galen, Rosemarie Conigliaro

**Affiliations:** 1 Albert Einstein College of Medicine; 2 Westchester Medical Center

**Keywords:** lumbar puncture, simulation training, internal medicine, medical education

## Abstract

This article was migrated. The article was not marked as recommended.

**Background:** Lumbar puncture (LP), although not mandatory for internal medicine (IM) residents to perform, remains a vital procedure for hospitalized patients. The optimal method for training residents in LP is not established.

**Objective:** We implemented, and evaluated a curriculum (online video, post-video quiz, group discussion, checklist, simulation training) for training PGY-2 IM residents in performing LPs.

**Methods:** We surveyed residents after completion of the curriculum and compared LP logs for the cohort who participated in this training to the prior year of residents without the curriculum.

**Results:** Overall survey response rate was 65%. 98% of residents found the pre-course materials useful; 92% agreed or strongly agreed that the in-person training session helped them to correctly perform an LP. 90% of residents found simulation training useful and 84% responded that the training session increased their confidence to perform an LP. However, compared to the prior residency class who did not have LP training, the initial class that underwent training did not perform more LPs during their residency: median 2 (inter-quartile range 0-5) vs. 2 (inter-quartile range 0-4.25), respectively (p = 0.98).

**Conclusion:** Despite not leading to an increase in LP rates, our curriculum was very well received by PGY-2 IM residents.

## Introduction

In 2006, the American Board of Internal Medicine (ABIM) reduced the number of procedures required by internal medicine (IM) residents for board eligibility, eliminating lumbar puncture (LP) as one of these ( Sacks et al. 2017). This revision reflected a shift away from prioritizing performing bedside procedures in IM residency based on the perceived decline in practicing internists performing these procedures ( Wigton et al. 2007). However, the need for these procedures on the inpatient services and intensive care units has not gone away. LP remains an important diagnostic procedure, critical in the evaluation of potentially life-threatening neurologic conditions and infectious diseases. As the number of IM trainees and supervising attending physicians with lumbar puncture skills has decreased, patients who require these procedures may experience delays in care. Internal medicine teams may rely on other services, such as neurology or neuroradiology, to perform lumbar punctures for their patients.

In recent years, there has been renewed interest in performing and training in bedside procedures in IM in conjunction with the rise of Hospital Medicine. One factor that has driven the renewal of bedside procedures has been the incorporation of ultrasound-guidance for bedside procedures in IM, as part of the point-of-care ultrasound (POCUS) movement ( Bhagra et al. 2016). POCUS has been evaluated as an aide in performing the LP procedure, but this is an advanced skill not yet in widespread use and was not utilized in this curriculum ( Soni et al. 2016). Determining the best/optimal way to incorporate training for bedside procedures, such as lumbar puncture, in residency is an area in need of more research ( Henriksen et al. 2017).

Given increased interest in the subject of bedside procedures such as LP, new curricula are needed to meet the demand. These curricula must also be properly evaluated in order to make key decisions regarding allocation of time for training, given the limits to clinical training time. Simulation-based mastery learning, a time and resource intensive training program, has been demonstrated to improve IM residents’ short-term LP skills, but the long term impact of this LP curricula has not been evaluated ( Barsuk et al. 2012). We therefore designed, implemented, and evaluated a novel curriculum for training IM residents in LP and assessed its impact on resident performance of this procedure.

## Methods

**Table 1.** A curriculum for LP training in internal medicine residency: mandatory for all PGY-2 residents at the start of academic year

Prior to In-Person Session	Group Session (1.5 hours)
Watch NEJM Video	Group discussion of Quiz Results
Complete Post-video Quiz	Observe expert perform simulated LP using procedure checklist
Review procedure checklist	Practice on mannequin with real-time feedback from expert and peers

Our mandatory LP curriculum occurred during dedicated curricular time scheduled specifically for second-year IM residents ( Table 1). Using a flipped classroom approach, residents prepared for the in-person training by watching the New England Journal of *MedicineLP* video available via institutional subscription (Mile et al. 2006). Residents were required to complete a post-video quiz created by the authors (supplement 1: LP Post-Video Quiz). A validated checklist for the lumbar puncture procedure was adapted for local use and made available to the residents prior to the group session ( Berg et al. 2013). The 1.5-hour group session (approximately 10 residents per session) began with a review of the questions in the post-video quiz, including a discussion of wrong answer choices. Next, an expert in the procedure (B.T.G.) demonstrated the lumbar puncture technique using the checklist and a commercially available mannequin: the M43B Lumbar puncture simulator-II, Limbs & Things Ltd., Sussex Street, Bristol, UK ( Uppal at al. 2011; Lumbar Puncture and Epidural Simulator). Each resident then performed a simulated lumbar puncture on the mannequin following the checklist with real-time feedback on his or her technique with peer and expert feedback.

The curriculum was required for the 2016 and 2017 IM postgraduate year 2 (PGY-2) categorical cohorts in the fall of their second year at Montefiore Medical Center, a large urban tertiary care center in Bronx, NY. Residents completed an anonymous survey electronically following the group session. In addition, we collected LP logs for the 2016 cohort at the time of graduation (June 2018) and compared them to those of the 2015 cohort, the prior year’s trainees who did not receive this training. Both the median number of LPs performed and the proportion of residents performing at least 1 LP were calculated. The significance of differences in the groups was tested using rank-sum and chi-squared tests. We performed statistical analysis using GraphPad Prism version 7.0 software (GraphPad Software Inc., San Diego, CA). This study was evaluated by the Albert Einstein College of Medicine Institutional Review Board and was granted approval as a quality improvement initiative (IRB# 2018-8838).

## Results/Analysis

**Figure 1.**
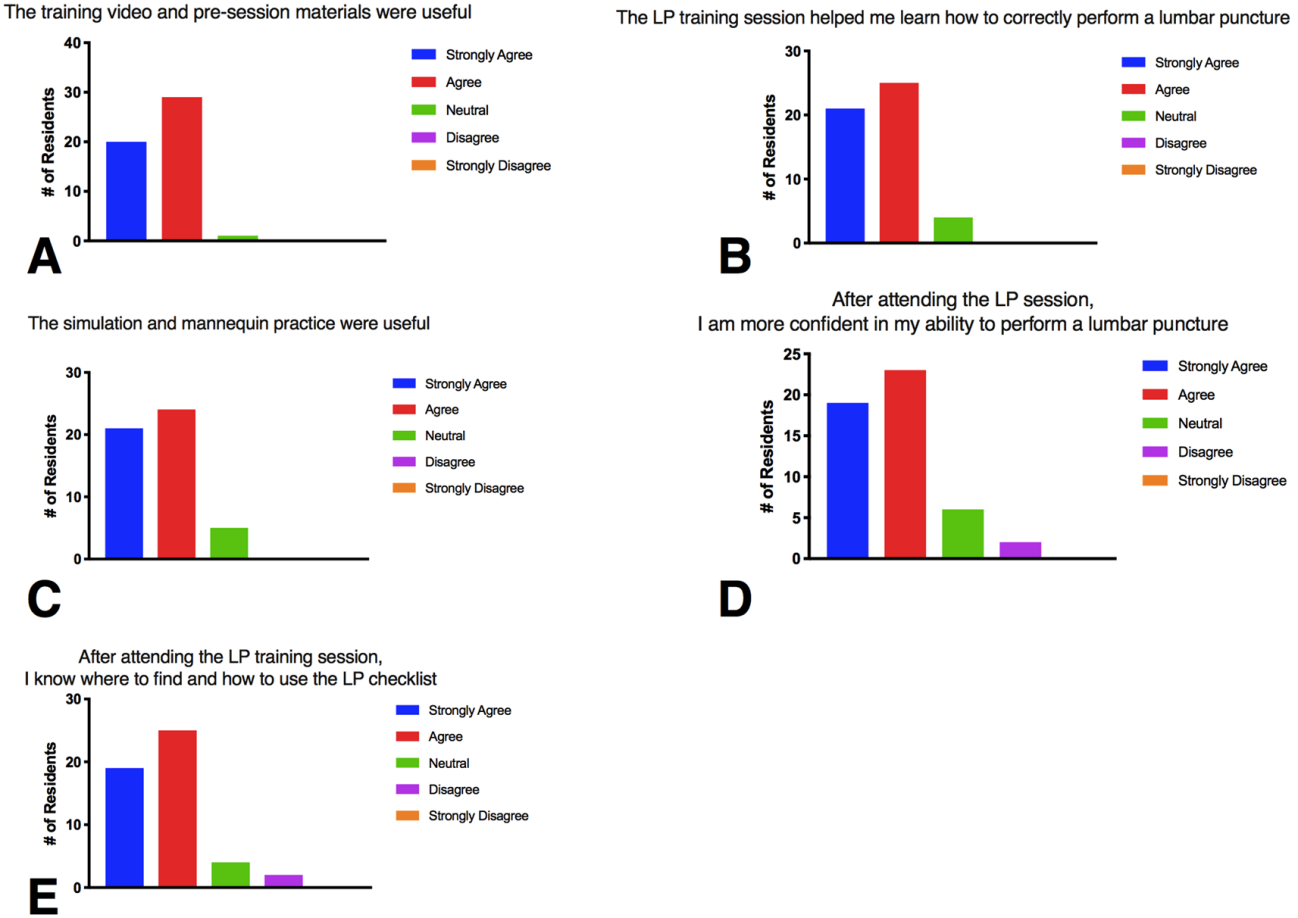
Resident survey results for two years of the LP curriculum (2016, 2107) (n = 50 respondents).

**Figure 2.**
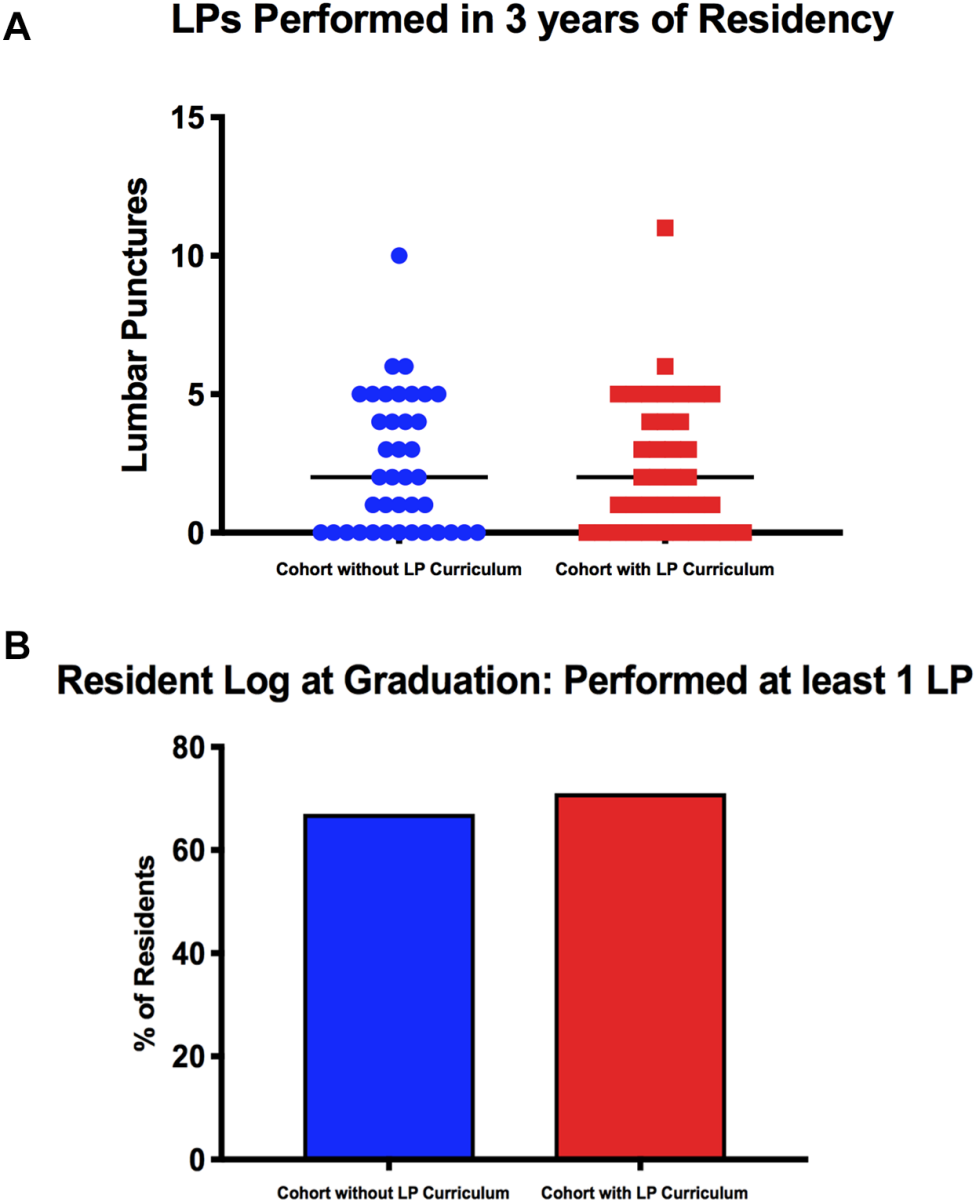
Resident LP logs at graduation. A) Scatter plot of resident-performed LPs at graduation for the 2017 graduation class (cohort without LP curriculum, n = 39) compared with the 2018 graduating class (cohort with LP curriculum, n =38). Points are values for individual residents; black lines represent median for each cohort (p = 0.98). B) Residents who did at least one LP by graduation: 66.7% vs. 71.1 % (p=0.68).

Combined results from two years of resident survey data are shown in Figure 1A-1E. The survey response rate was 62% in the 2016 year (n = 24) and 68% in the 2017 year (n= 26), for a total of 50 responses. 98% of residents agreed or strongly agreed that the pre-course materials were useful (1A). 92% of residents agreed or strongly agreed that the in-person training session (review of quiz, simulation training) helped them to correctly perform an LP (1B). 90% of residents agreed or strongly agreed that the simulation training was useful and 84% of residents agreed or strongly agreed that the training session increased their confidence in their ability to perform an LP (1C, 1D). 88% of residents responded that they knew how to use the LP checklist after attending the session (1E).

The training did not lead to an increase in median number of LPs performed by residents at graduation. The 2015 PGY-2 cohort who graduated in 2017 (the group which did not have the curriculum) logged a median of two LP’s per resident (with inter-quartile range 0-5) and the 2016 cohort (the first class that underwent training and graduated in 2018) logged a median of two LP’s per resident (with inter-quartile range 0-4.25) (p = 0.98) ( Figure 2A). The percentage of residents who did at least one LP during residency did not change significantly with exposure to the LP curriculum (p=0.68), 66.7% vs. 71.1%, respectively ( Figure 2B). Approximately 25% of our residents logged five or more LPs and there was no significant change with implementation of the LP curriculum.

## Discussion

The relative infrequence, but critical importance of lumbar puncture performance poses a unique challenge for training, which prompted this study evaluating a novel LP curriculum. While studies of simulation training for LP have been conducted in pediatric and radiology residency training programs, few studies have examined the value and impact of LP simulation training for IM residents ( McMillan et al. 2016; Kessler et al. 2011; Faulkner et al. 2015). While a prior study by Barsuk, et al., evaluated the immediate benefits of LP simulation-based education of IM residents, to our knowledge no studies have assessed a curriculum’s impact on IM residents’ real-life performance of this procedure ( Barsuk et al. 2012). Our study demonstrates that despite the ABIM’s elimination of performing an LP as a certification requirement, it is still done by approximately 70% of internal medicine residents at an urban, academic medical center at least once during their residency. Any future revision to the ABIM certification policies surrounding this procedure should take these findings into account.

A curriculum utilizing a partially flipped classroom approach, group discussion, and simulation training was overwhelmingly well received by PGY-2 IM residents. The curriculum was not only perceived to be valuable, but also led to increased confidence in residents’ ability to perform the procedure. Given our findings, internal medicine programs should consider devoting curricular time to lumbar puncture training.

We hypothesized that this LP curriculum would encourage residents to do LPs on their own patients expeditiously, rather than consulting neurology or referring patients to neuroradiology. However, by comparing resident logs at graduation before and after the mandatory curriculum was implemented we did not find a significant increase in the number of residents who performed at least one LP during their residency. The average number of LPs per resident also remained unchanged. Given the infrequent requirement for LP, it is possible that these outcomes are not the appropriate measure of the success of our curriculum. We do not know if our curriculum led to more successful and safer LPs (e.g., with fewer attempts or fewer complications), as these outcomes were not evaluated. Furthermore, it is possible that our curriculum raised awareness about indications and the importance of LP, encouraging residents to attempt them, albeit unsuccessfully, on the inpatient services; we do not have data on instances in which an LP attempt was unsuccessful at the bedside and therefore required the expertise of another service.

Our residency program requires that five LPs be successfully completed for “certification” to perform an LP independently; approximately 25% of our residents logged five or more LPs, with no statistically significant change after the LP curriculum was implemented. It is possible that residents stop logging once they reach the 5 LP requirement/mark, but this under-reporting is likely to have affected all cohorts equally (i.e., before and after the curriculum).

One limitation of this study is its reliance on resident self-report to support the value of our LP curriculum, rather than objective measures of resident skill at performing the procedure. Additional limitations include the availability of an expert in this procedure to conduct in-person training and resources to purchase the mannequin for simulation training. Further work should include assessment of residents’ LP skills before and after simulation training, as well as longitudinal follow up to determine retention of the material and skills.

## Conclusion

Additional work is needed to evaluate the optimal way to train internal medicine residents in the necessary skill of bedside lumbar puncture. While residents rated our curriculum highly, it did not significantly increase resident performance of the procedure. A required curriculum composed of readily available didactic materials, group discussion, procedure checklist, and simulation training might play a role in internal medicine resident LP training.

## Take Home Messages

• Most Internal Medicine residents at a large, urban medical center in the United States perform lumbar puncture during their residency.
• A simulation-based, partly flipped classroom curriculum for lumbar puncture training was well received by PGY-2 internal medicine residents.

## Notes On Contributors

Benjamin T. Galen, BS, MD, Assistant Professor of Medicine, Albert Einstein College of Medicine, Associate Program Direction and Director of Ultrasound and Procedure Training, The Einstein/Montefiore Internal Medicine Residency Program. https://orcid.org/0000-0001-8172-258X

Rosemarie L. Conigliaro, BA, MD, Westchester Medical Center, Section Chief, General Internal Medicine, Vice Chair of Education, Department of Medicine, Professor of Clinical Medicine, New York Medical College.
