# Supplementary material for: A Curriculum for Lumbar Puncture Training in Internal Medicine Residency
Source: MedEdPublish (2016). 2019 Feb 26;8:33. [Version 1] doi: 10.15694/mep.2019.000033.1 (PMC10712592; doi:10.15694/mep.2019.000033.1)
Supplement: Peer review history

# Reviewer report: Reviewer response for version 1

**DOI:** 10.21956/mep.19776.r29714

**Published:** 2019-06-21

**Authors:** Hassaan Waqar

**Affiliations:** 1 St Helens and Knowsley Teaching Hospitals NHS Trust – Lead Employer

This review has been migrated. The reviewer awarded 3 stars out of 5 Development of a curriculum for an important and relevant skill such as lumbar puncture is an important area of research. This article shows a structured method of introducing a curriculum for performing this skill and reviews the effectiveness of the curriculum. The number of lumbar punctures performed may not be an accurate measure of the utility of such a curriculum and can be influenced by a number of factors. The take home messages section was quite brief and could have featured more points.

Reviewer Expertise:

NA

No decision status is available

# Reviewer report: Reviewer response for version 1

**DOI:** 10.21956/mep.19776.r29716

**Published:** 2019-02-28

**Authors:** Balakrishnan Nair

**Affiliations:** 1 Centre for Medical Professional Development and University of Newcastle

This review has been migrated. The reviewer awarded 3 stars out of 5 Doing a lumbar puncture is a necessary skill ; it should be necessary part of any internal medicine training program in my view. Even though infrequently done now, , when indicated , it should be done without delay to make a prompt diagnosis , which could be life saving . So this study on a flipped class room approach followed by log book review is timely It would have been useful to see the difference in the quality , attempts and feedback from patients . I am sure the authors will be contemplating this follow up as mentioned in the discussion

Reviewer Expertise:

NA

No decision status is available

# Reviewer report: Reviewer response for version 1

**DOI:** 10.21956/mep.19776.r29713

**Published:** 2019-02-27

**Authors:** P Ravi Shankar

**Affiliations:** 1 American International Medical University

This review has been migrated. The reviewer awarded 3 stars out of 5 Thank you for the opportunity to review this interesting manuscript. The authors evaluated a module for training internal medicine residents to perform lumbar puncture. The methodology used has been well described. The authors used a flipped classroom approach and residents participated actively in the learning process. Appropriate ethical permissions were obtained. I am not familiar with Residency requirements in the United States but find it rather strange that lumbar puncture was removed from the list of certifiable skills for Internal Medicine. The authors mention that lumbar puncture is performed relatively infrequently. Has the number of lumbar punctures carried out decreased because of the availability of other diagnostic modalities? In many countries lumbar puncture as a diagnostic technic is learned during the undergraduate medical program and remains an important diagnostic procedure. How many residents participated in the educational module and what percentage of them completed the questionnaire? The authors have evaluated resident feedback using a Likert scale. Obtaining free text comments and reflections of the residents will also be useful.

Reviewer Expertise:

NA

No decision status is available

# Reviewer report: Reviewer response for version 1

**DOI:** 10.21956/mep.19776.r29715

**Published:** 2019-02-26

**Authors:** Trevor Gibbs

**Affiliations:** 1 AMEE

This review has been migrated. The reviewer awarded 3 stars out of 5 Thank you for asking me to review this paper. The issue of what constitutes a learning outcome for graduates of a medical school is very dependent upon the needs of new graduates, taken in the context of the country or region the school is based in. Hence it is a variable. However I do feel that this is an important topic.I appreciated the creation of various learning approaches within the LP course that the authors put together- a great example of creating a toolbox of approaches, all of varying activity levels. I was not therefore surprised by the learners positive responses.Ifeel however that the authors missed a chance to evaluate this combined approach and individual techniques by only carrying out a low level Kirkpatrick evaluation. As the authors addressed it would have been better / idea if they had waited a short period of time and asked the students to reflect upon the teaching and its usefulness to the rral world. I fail to see the value of measuring the number of LPs performed because there are so many conflicting reasons that can affect this.Perhaps a greater explanation of the individual teaching methods and why they were chosen might have helped this paper.

Reviewer Expertise:

NA

No decision status is available
